# Influence of Environmental Factors on Seasonal Variation in Plankton Dynamics in Nearshore Waters of Eastern Guangdong, China

**DOI:** 10.3390/plants15162532

**Published:** 2026-08-21

**Authors:** Santhoshkumar Chinnappan, Hongyan Xiang, Zhuoming Xiao, Keying Cheng, Wenhua Liu, Ping Li

**Affiliations:** 1 Guangdong Provincial Key Laboratory of Marine Biotechnology, Shantou University, Shantou 515063, China; san.c175@yahoo.co.in (S.C.);; 2 International Joint Research Center for Marine Ecological Protection and Disaster Prevention, Shantou University, Shantou 515063, China; 3 College of Fisheries, Guangdong Ocean University, Zhanjiang 524088, China

**Keywords:** redundancy analysis, plankton community, zooplankton abundance, environmental factors, phytoplankton abundance, seasonal variations

## Abstract

Phytoplankton and zooplankton are critical basal resources in regulating aquatic material and energy fluxes. We investigated how environmental variables shape seasonal plankton abundance and diversity across nearshore zones in Eastern Guangdong, China. We identified 62 phytoplankton taxa (dominated by Bacillariophyceae at 88.52%) and 34 zooplankton species (dominated by calanoid copepods at 36.36%). Redundancy analysis (RDA) demonstrated that seasonal shifts in temperature, salinity, pH, dissolved oxygen, and dissolved nutrients (N, P, Si) significantly drive spatial community variability (Y ≥ 0.02). Dominant bioindicator species exhibited strong seasonal ties to environmental gradients; the diatom *Skeletonema costatum* strongly correlated with inorganic nitrogen during autumn, while the copepod *Acartia centrura* was heavily influenced by summer salinity and nutrient loads. Overall, phytoplankton diversity was higher than zooplankton diversity. These baseline findings regarding dominant copepods, diatoms, and eutrophic field parameters offer valuable insights for developing live feed alternatives in aquaculture and identifying bioactive substances for pharmaceutical applications.

## 1. Introduction

Nearshore environments are critical transitional zones linking marine and freshwater ecosystems, facilitating a dynamic exchange of energy and matter [1]. Due to their proximity to urban and industrial centers, nearshore waters are highly vulnerable to anthropogenic disturbances. Rapid urbanization and coastal industrial development have increased the discharge of land-based chemical runoff, posing significant risks to both human and environmental health [2,3]. While chemical monitoring provides critical exposure data, tracking every synthetic substance discharged into coastal zones remains logistically impractical [4]. Consequently, implementing a reliable, biologically grounded approach is essential for assessing the ecological health of nearshore systems.

Phytoplankton and zooplankton play a fundamental role in regulating the marine biological pump and global biochemical cycles. Zooplankton maintains the stability of coastal food-webs by serving as a primary energetic conduit between autotrophic producers and higher trophic levels. Conversely, while phytoplankton constitute less than 1% of global photosynthetic biomass, they drive approximately 50% of global net primary production [5]. However, plankton community dynamics are tightly driven by localized physicochemical and biological fluctuations [6]. Plankton assemblages in nearshore environments are therefore highly sensitive to shifting water quality mediated by changing seasons and anthropogenic resource inputs.

Plankton dynamics serve as excellent ecological indicators for water quality monitoring. They are closely coupled to environmental transitions and respond to localized disturbances non-linearly, making them highly sensitive to subtle environmental shifts [7,8]. Previous research suggests that marine plankton variations can offer a more sensitive reflection ecosystem stress than conventional isolated water-chemistry measurements [9,10]. In the northern South China Sea and adjacent Guangdong coastal waters, historical baseline studies demonstrated that seasonal monsoon transitions, freshwater pulses from the Hanjiang River estuary, and localized upwelling intensities directly regulate plankton biomass, causing rapid shifts between diatom-dominated and dinoflagellate-dominated states [11,12]. Understanding these underlying drivers within specific subtropical nearshore spaces is crucial for establishing reliable biodiversity indicators for coastal zone management.

The relationships between dominant species and environmental variables—such as temperature, salinity, pH, dissolved oxygen, and dissolved inorganic nutrients—can be robustly modeled using multivariate statistical methods. Redundancy analysis (RDA) is widely preferred for these ecological datasets, as it is assumes a short environmental gradient and effectively accommodates low species turnover across seasonal sampling stations. Operating on a constrained linear model, RDA evaluates the relative explanatory power of individual environmental parameters in accounting for variations in species composition. In contrast, while canonical correlation analysis and principal component analysis function linearly, they do not partition the specific explanatory importance of environmental variables against the observed variation in biological community structures [13]. Unconstrained non-linear models, such as correspondence analysis and detrended correspondence analysis, can also introduce interpretation challenges when isolating direct, causal relationships between target hydrographic variables and specific plankton diversity indices.

The seasonal succession of phytoplankton and zooplankton in relation to complex hydrographic shifts within the anthropogenically influenced nearshore waters of Eastern Guangdong remains poorly characterized. To address this gap, we monitored seasonal plankton dynamics across nearshore zones in Eastern Guangdong, China. Using conventional morphological identifications and quantitative counting alongside spatial water chemistry analyses, this study aimed to: (i) characterize seasonal plankton community structures and assemblages, (ii) identify the key environmental variables driving the seasonal distribution of phytoplankton and zooplankton, and (iii) quantify the exact variance in plankton abundance explained by localized environmental parameters. These findings contribute to a mechanistic understanding of plankton dynamics in subtropical coastal ecosystems and support the development of targeted conservation strategies for marine biodiversity.

## 2. Results

### 2.1. Spatiotemporal Variations in Local Environmental Variables

**Figure 1:**
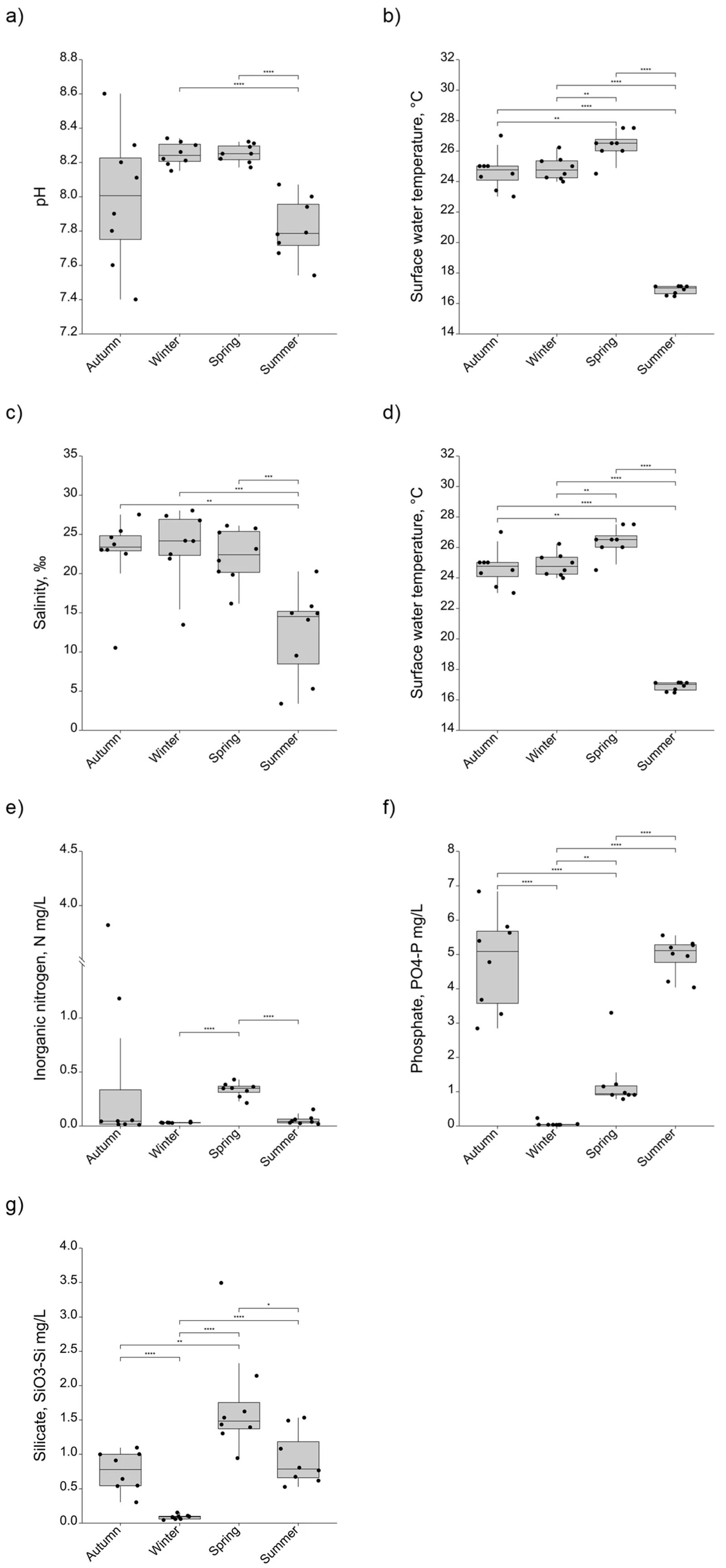
Seasonal variation in local environmental variables: (**a**) pH, (**b**) surface water temperature, (**c**) salinity, (**d**) dissolved oxygen, (**e**) inorganic nitrogen content, (**f**) phosphate content, and (**g**) silicate content in water samples collected from nearshore waters in Eastern Guangdong, China. Asterisks denote statistically significant differences between the two groups: **** *p* = 0.001, *** *p* = 0.02, ** *p* = 0.01 and * *p* = 0.05.

Local environmental parameters were monitored in nearshore water samples during four seasons (Figure 1). Dissolved oxygen levels were highest (6.44 ± 0.27 mg/L) in winter and statistically significant (*p* < 0.01). The lowest levels of dissolved oxygen (4.22 ± 2.04 mg/L), pH (7.82 ± 0.18), water temperature (16.9 ± 0.29 °C), and salinity (12.3 ± 5.7 ‰) were found in summer. Winter salinity (23.5 ± 4.65 ‰) was nearly twice that of summer but not statistically significant compared to autumn. Inorganic nitrogen, phosphorus, and silicate concentrations were lowest in winter, silicate and inorganic nitrogen were highest in spring, and phosphorus was highest in summer and autumn, suggesting seasonal changes in local environmental variables.

Samples were grouped based on proximity to the three distributaries of Hangjiang River: Lianyang River (S1, S2), Waisha River (S3, S4, S5), and Xinjin River (S6, S7, S8). Winter dissolved oxygen levels in samples from Xinjin River were significantly higher (*p* = 0.02) than those from Lianyang River. Statistically significant differences were observed between the Lianyang and Waisha rivers in spring (*p* = 0.02), summer (*p* = 0.001), and autumn (*p* = 0.05) (SM1, https://doi.org/10.6084/m9.figshare.21997712). No significant variations were noted for salinity, silicate, phosphorus, and nitrogen.

### 2.2. General and Assemblage Species Compositions

The functional groups of phytoplankton and zooplankton in aquatic environments can vary based on nutrient content. In this study, 96 species of phytoplankton were identified, with 62 from the Bacillariophyceae (centric and pennate diatoms), Dinophyceae, Chlorophyceae, Cyanobacteria, and Conjugatophyceae groups. Diatoms dominated phytoplankton composition, followed by dinoflagellates. The dominant phytoplankton genera in autumn and winter were *Cyclotella* and *Coscinodiscus* (Table 1), with only Coscinodiscus present in summer. Four species, *Ditylum brightwellii*, *Thalassionema nitzschioides*, *Thalassiothrix longissima*, and *Skeletonema costatum*, were dominant across all seasons, along with *Ditylum sol*. A total of 34 zooplankton species were recorded, with the ten classes being Calanoid, Choreotrichida, Cyclopoida, Harpacticoida, Poecilostomatoida, Decapoda, Spinicaudata, Arcellinida, Diplostraca, and Gastropoda. Maximum zooplankton diversity was seen in summer. *Favella* sp., *Paracalanus parvus*, and copepod larvae dominated autumn, while copepod larvae and *Paracalanus parvus* dominated winter and spring, and *Tintinnopsis radix* dominated summer (Table 1 and Table 2).

#### 2.2.1. Influence of Environmental Variables on Phytoplankton Distribution and Diversity Indices

**Figure 2:**
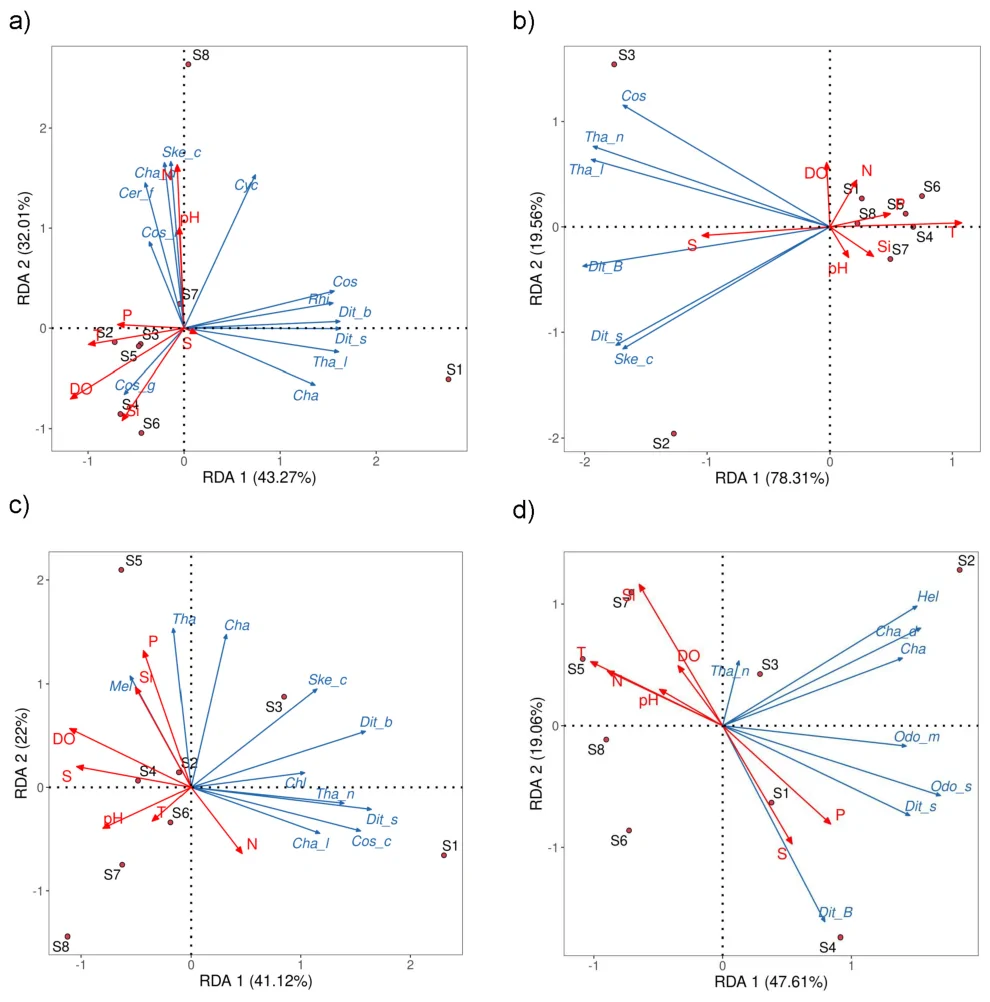
Redundancy analysis (RDA) illustrating the relationship between environmental variables and dominance of phytoplankton ((**a**) autumn, (**b**) winter, (**c**) spring, and (**d**) summer). The letters with black dots represent the dominant species of *Skeletonema costatum* (Ske_c), *Cyclotella* sp. (Cyc), *Chaetoceros decipiens* (Cha_d), *Ceratium furca* (Cer_f), *Coscinodiscus radiates* (Cos_r), *Coscinodiscus granii* (Cos_g), *Coscinodiscus* sp. (Cos), *Coscinodiscus centralis* (Cos_c), *Rhizosolenia* sp. (Rhi), *Thalassiothrix longissima* (Tha_l), *Ditylim sol* (Dit_S), *Ditylum brightwellii* (Dit_b), *Melosira* sp. (Mel), *Thalassionema nitzschioides* (Tha_n), *Thalassiothrix longissima* (Tha_l), *Chaetoceros* sp. (Cha), and *Chlorella* sp. (Chl); and the letters with red arrows represent the local environmental variables: dissolved oxygen (DO), salinity (S), temperature (T), silicate (Si), inorganic nitrogen (N), phosphate, and pH.

Redundancy analysis (RDA) was performed to evaluate how environmental variables correlate with seasonal phytoplankton distribution (Table 3 and Figure 2). During autumn, *Skeletonema costatum* (Ske_c) and *Chaetoceros decipiens* (Cha_d) exhibited a strong relationship with inorganic nitrogen, whereas Cyclotella sp. showed only a moderate correlation at stations S7 and S8 (Figure 2a). Taxa such as *Ceratium furca* (Cer_f), *Coscinodiscus* sp. (Cos), *Coscinodiscus radiates* (Cos_r), *Coscinodiscus granii* (Cos_g), and *Chaetoceros* sp. (Cha) were associated with low inorganic nitrogen, phosphate, and temperatures at sites S2, S3, S4, S5, and S6. Meanwhile, *Rhizosolenia* sp. (Rhi) and *Thalassiothrix longissima* (Tha_l) were influenced by salinity at S1. In winter, *Ditylum brightwellii* (Dit_b) was strongly influenced by salinity, whereas species including *Ditylum sol* (Dit_s), *Skeletonema costatum* (Ske_c), *Thalassiothrix longissima* (Tha_l), *Thalassionema nitzschioides* (Tha_n), and *Coscinodiscus* sp. (Cos) were moderately influenced at stations S2 and S3 (Figure 2b). A positive correlation was observed between phytoplankton abundance and silicate (r = 0.762 *). In spring, *Melosira* sp. (Mel), *Thalassiothrix longissima* (Tha_l), and *Chaetoceros* sp. (Cha) displayed a strong connection to silicate concentrations at S2 and S4. Conversely, *Coscinodiscus centralis* (Cos_c), *Ditylum sol* (Dit_s), and *Thalassionema nitzschioides* (Tha_n) were linked to inorganic nitrogen at S1 (Figure 2c). During summer, *Ditylum brightwelliii* (Dit_B) responded strongly to salinity, while *Ditylum sol* (Dit_s), *Odontella sinensis* (Odo_s), and *Odontella mobiliensis* (Odo_m) were related to phosphate levels and moderate salinity at stations S1 and S4 (Figure 2d).

**Figure 3:**
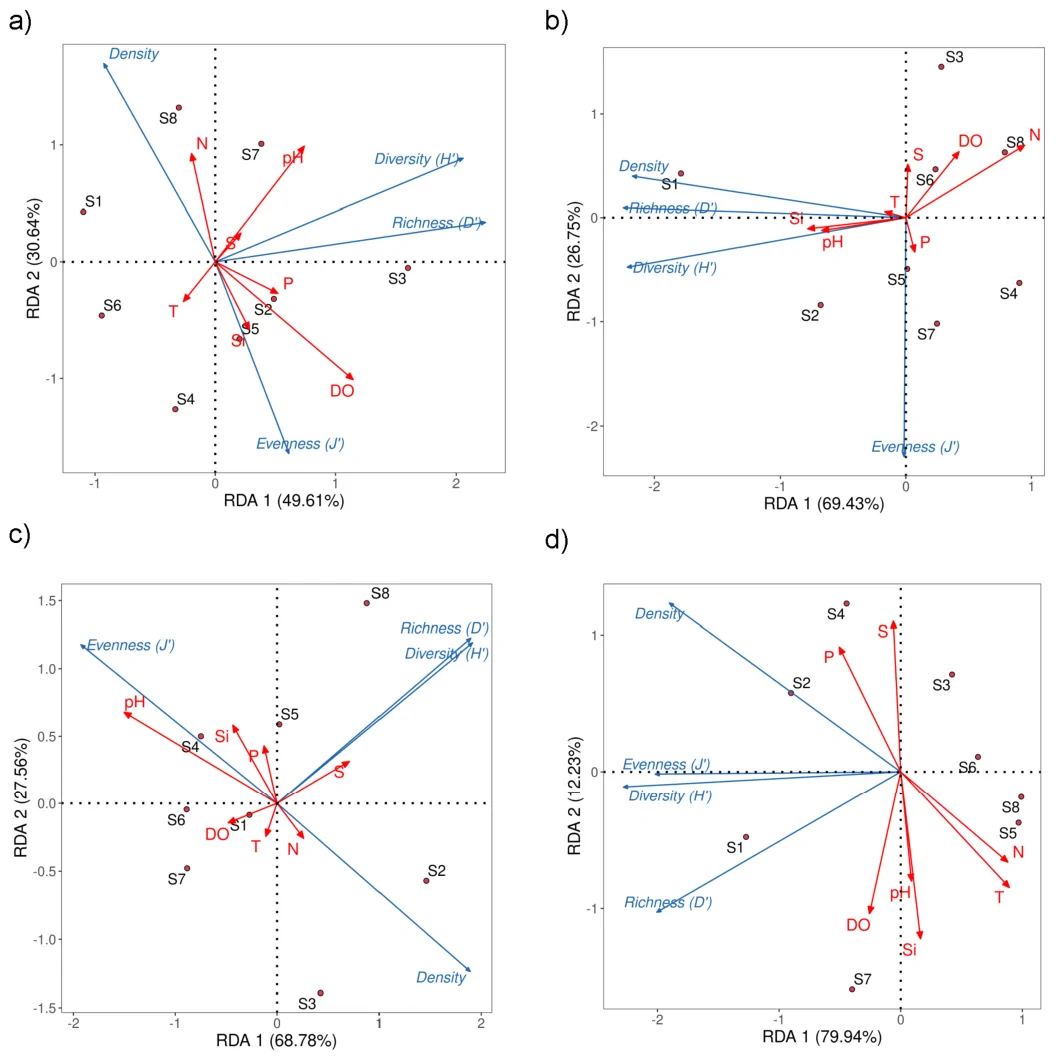
Redundancy analysis (RDA) illustrating the relationship between environmental variables dominance of zooplankton ((**a**) autumn, (**b**) winter, (**c**) spring, and (**d**) summer). The letters with black dots represent the dominant species of *Tintinnopsis* sp. (Tin), *Favella* sp. (Fav), *Oithona similis* (Oit_s), *Paracalanus parvus* (Par_p), *Oithona brevicornis* (Oit_b), *Acrocalanus* sp. (Acr), *Acrocalanus gibber* (Acr_g), *Penilia* sp. (Pen_s), *Microsetella norvegica* (Mic_n), *Tintinnopsis radix* (Tin_r), Polychaeta larva (Pol_l); and the letters with red arrows represent the local environmental variables: dissolved oxygen (DO), salinity (S), temperature (T), silicate (Si), inorganic nitrogen (N), phosphate, and Ph.

These findings indicate that environmental parameters—specifically inorganic nitrogen, phosphate, silicate, salinity, temperature, and pH—play a key role in shaping the phytoplankton community. These variables also affected the richness (D), evenness (J), and diversity (H) values of the community (Figure 3, Table 3). RDA3 and RDA4 were excluded from the discussion due to their low explanatory proportion (Table 2). In autumn, diversity (1.028, 0.442) and richness (1.121, 0.169) positively influenced the correlation of phytoplankton species with salinity and pH at S7, while displaying a negative correlation with temperature. Evenness was shaped by silicate, phosphate, and dissolved oxygen at S2 and S5, whereas population density was dominated by nitrogen at site S8. The cumulative contribution of RDA1 and RDA2 to the species–environment relationship in autumn was 80.25% (Figure 3a). In winter, density (−1.087, 0.201), richness (−1.123, 0.048), and diversity (−1.107, 0.238) were tied to silicate, temperature, and pH at S1 and S2, while evenness values are related to phosphate at S7 and S4. The total cumulative contribution of the RDA1 and RDA2 for this season reached 96.18% (Figure 3b). In spring, diversity (0.957, 0.595) and richness (0.948, 0.612) positively influenced salinity dynamics at S5 and S8, while evenness was driven by pH, silicate, and phosphate at S4. Density (0.945, −0.618) was linked with inorganic nitrogen at S3 and S4, with RDA1 and RDA2 combining for a cumulative contribution of 96.34% (Figure 3c). Finally, the summer season showed observed diversity (−0.951, 0.616) and richness (−1.002, −0.513) trends with slight enhancements tied to dissolved oxygen, pH, and silicate at S1 and S7. Density (−0.951, 0.616) influenced phosphate and silicate at sites S2 and S4, whereas temperature and nitrogen showed negative concentrations at S5 and S8. The cumulative contribution of the RDA1 and RDA2 in summer was 92.17% (Figure 3d).

#### 2.2.2. Influence of Environmental Variables on Zooplankton Distribution

**Figure 4:**
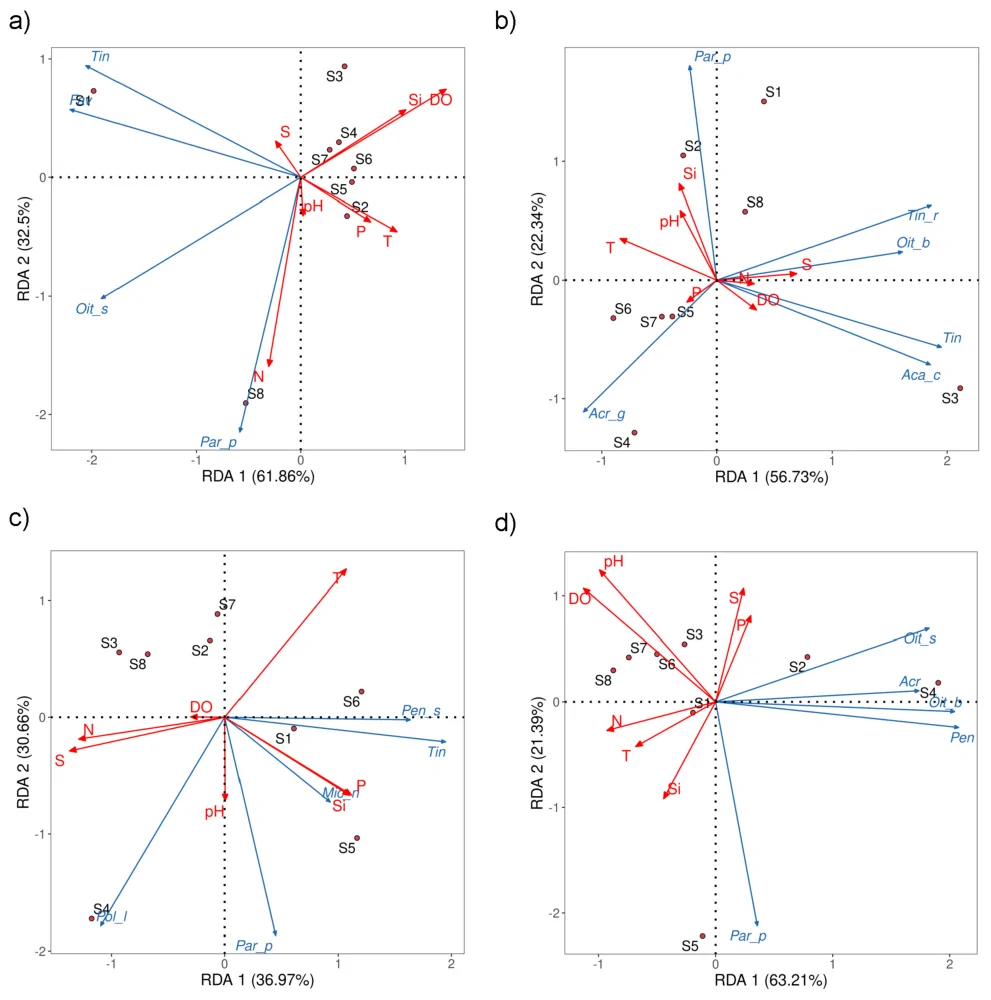
Redundancy analysis (RDA) examines the association between environmental parameters and phytoplankton richness, evenness, diversity, and density ((**a**) autumn, (**b**) winter, (**c**) spring, and (**d**) summer). The letters in black and red represent the physiochemical parameters of dissolved oxygen (DO), salinity (S), surface water temperature (T), silicate (Si), inorganic nitrogen (N), phosphate (P), and pH.

In autumn, *Tintinnopsis* sp. (Tin) and *Favella* sp. (Fav) were linked to salinity, while *Paracalanus parvus* (Par_p) relied on high inorganic nitrogen at S1 (Figure 4a). *Paracalanus parvus* (Par_p) depended on silicate and moderate temperature and pH at S2. *Oithona brevicornis* (Oit_b) and *Tintinnopsis radix* (Tin_r) were affected by salinity and low nitrogen at S8, while *Tintinnopsis* sp. (Tin) and *Acartia centrura* (Aca_c) were related to dissolved oxygen and moderate nitrate at S3. *Acrocalanus gibber* (Acr_g) was affected by low phosphate at S4, S5, S6, and S7 (Figure 4b). *Microsetella norvegica* (Mic_n) was strongly associated with phosphate and silicate at S1 and S5, while *Penilia* sp. (Pen_s) and *Tintinnopsis* sp. (Tin) were related to phosphate and moderate silicate and temperature at S1, S5, and S6 in the spring season (Figure 4c). During summer, *Oithona similis* (Oit_s), *Acrocalanus gibber* (Acr_g), and *Oithona brevicornis* (Oit_b) displayed considerable dependence on phosphate and salinity at S2 (Figure 4d).

**Figure 5:**
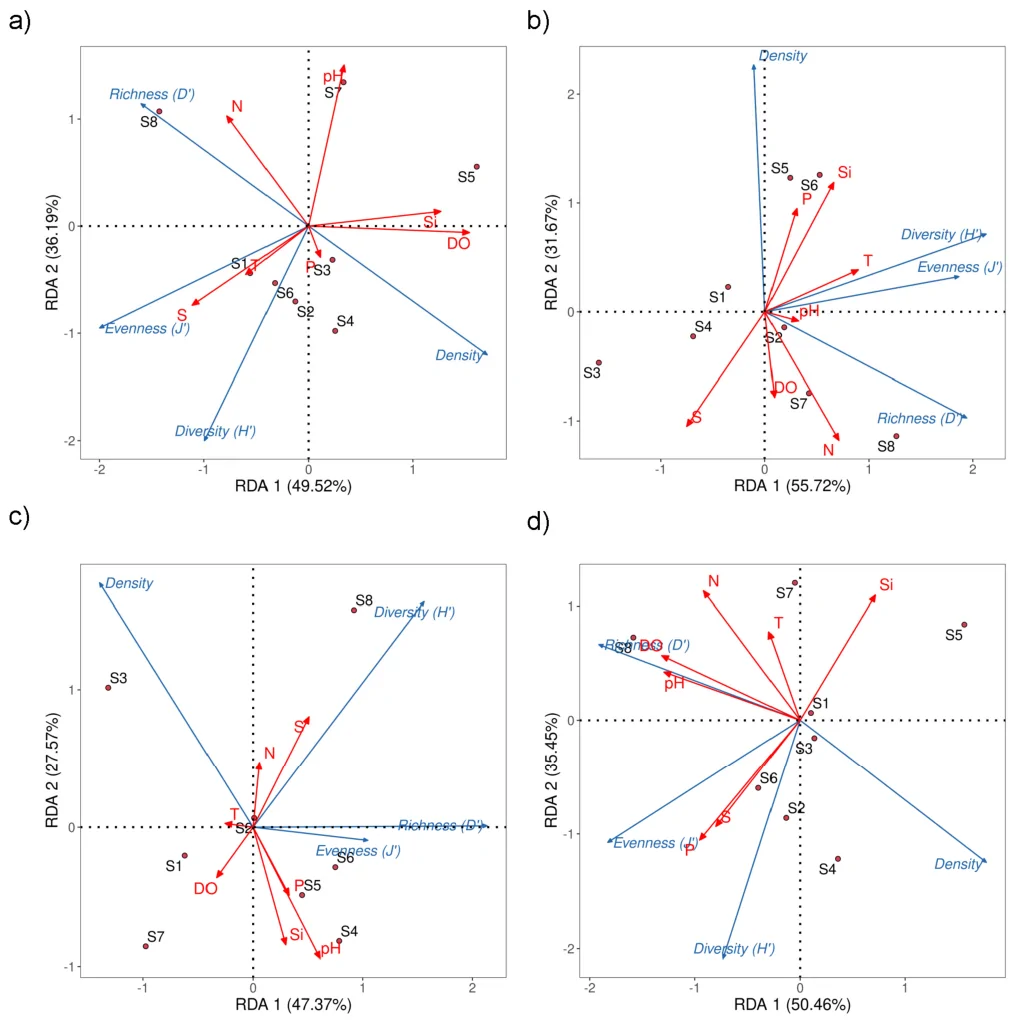
Redundancy analysis (RDA) examines the association between environmental parameters and zooplankton richness, evenness, diversity, and density ((**a**) autumn, (**b**) winter, (**c**) spring, and (**d**) summer). The letters in black and red represent the physiochemical parameters of dissolved oxygen (DO), salinity (S), surface water temperature (T), silicate (Si), inorganic nitrogen (N), phosphate (P), and pH.

**Figure 6:**
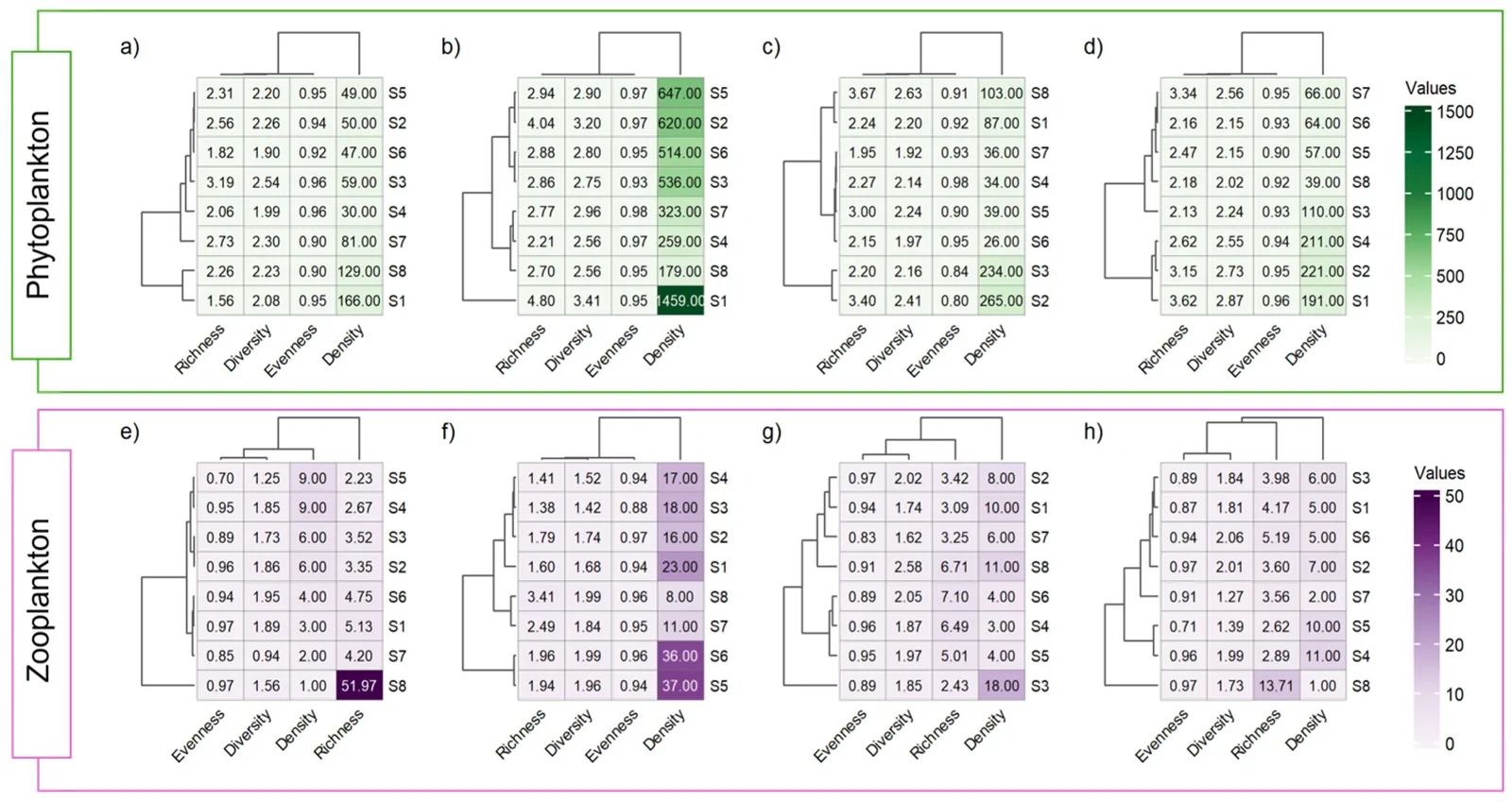
Ward cluster analysis of phytoplankton and zooplankton evenness, richness, diversity, and density in the Hanjiang River reflect the different sites and seasons. Eight sampling sites of phytoplankton ((**a**) autumn, (**b**) winter, (**c**) spring, and (**d**) summer) and zooplankton ((**e**) autumn, (**f**) winter, (**g**) spring, and (**h**) summer).

The influence of phosphate, dissolved oxygen, and inorganic nitrogen on the zooplankton species’ richness (D), evenness (J), density, and diversity (H) was apparent. In autumn, zooplankton richness (−0.800, 0.571) was correlated with nitrogen at site 8, whereas evenness (−0.999, −0.475) and diversity (−0.497, −1.000) were influenced by salinity and temperature at sites 1, 6, and 2. Density (0.856, −0.601) was negatively correlated with phosphate and dissolved oxygen at sites 3 and 4. The cumulative contribution of the zooplankton species and the environment variables was 85.71% (Figure 5a). In winter, environmental parameters, such as temperature, phosphate, and silicate, positively affected diversity (1.064, 0.357) and evenness (0.932, 0.161) at sites 5 and 6, and salinity negatively correlated at sites 3 and 4. Richness was (0.970, −0.485) related to dissolved oxygen and nitrogen at sites 2, 7, and 8. The cumulative contribution of the zooplankton species and the environment variables was 87.39% (Figure 5b). In spring, the higher diversity (0.779, 0.818) of zooplankton species was positively influenced by salinity and nitrogen at site 8, whereas the dissolved oxygen negatively correlated at sites 1 and 7. Evenness was (0.523, −0.047) correlated with phosphate, silicate, and pH at sites 4, 5, and 6. The cumulative contribution of the zooplankton species and the environment variables was 74.94% (Figure 5c). During summer, temperature, nitrogen, dissolved oxygen, and pH moderately impacted the richness (−0.956, 0.332) of zooplankton species at sites 7 and 8. Diversity (−0.364, −1.042) and evenness (−0.914, −0.537) were correlated with salinity and phosphate at sites 2 and 6. The cumulative contribution of the zooplankton species and the environmental variables was 85.91% (Figure 5d). RDA plots and cluster analysis (Figure 6) revealed higher density and richness values. Overall, environmental parameters affected the richness, evenness, density, and diversity of zooplankton species in the RDA plots and cluster analysis.

## 3. Discussion

Our correlation analyses revealed that localized physiochemical parameters significantly account for the overall diversity, dominance, and structural configuration of the plankton communities. This supports the hypothesis that hydrographic variables primarily regulate the diversity and structural stability of plankton networks in the nearshore waters influenced by the Hanjiang River. Across all seasons and spatial zones, the dominant taxonomic groups remained relatively consistent. The diatom Bacillariophyceae formed the vast majority of the phytoplankton assemblage (Table 1), whereas copepods heavily dominated the zooplankton community across all monitored sampling sites (Table 2). This structural dominance indicates that seasonal nutrient inputs, potentially driven by regional riverine discharges and agricultural irrigation runoff, selectively enrich fast-growing, opportunistic species, thereby steering regional community successions [14]. Systematically, nearshore plankton diversity and stability are collectively steered by a shifting baseline of temperature, pH, salinity, dissolved oxygen, and dissolved inorganic nutrients (nitrogen, phosphate, and silicate).

### 3.1. Effect of Local Environmental Factors on Phytoplankton Dynamics

The nutritional parameters had a significant impact on the plankton community, although their quantified contribution was relatively smaller than that of the physical and chemical parameters. This could be due to the planktonic community’s delayed response to changes in nutrient parameters [15]. The taxonomic assemblage and diversity of phytoplankton varied seasonally and between stations, as revealed by RDA ordination, which compared dominant species and environmental parameters. The composition and abundance of the phytoplankton community are primarily influenced by the concentration of nutrients [16]. The ability of *Skeletonema costatum* to tolerate high nitrate levels [17] and its growth in offshore waters was greatly accelerated by silicate [18]. *Skeletonema costatum* commonly dominates the diatom abundance in coastal waters [15,19,20]. Temperature is one of the most important environmental factors and influences the phytoplankton growth rate, cell size, biochemical structure, and nutritional needs [21]. Temperature differences due to seasonal changes are a significant factor affecting plankton diversity and stability [15]. In the spring, sites 2 and 8 had the highest phytoplankton diversity and richness, which was influenced by salinity. Salinity is a limiting factor of living organisms, and evaporation is most likely to affect the faunal and floral distributions of coastal ecosystems [22]. Silicate was the most important environmental factor influencing the phytoplankton distribution during spring and is one of the essential nutrients that regulate the phytoplankton distribution of biota in ecosystems and dominated diatoms with silicate concentration [23,24,25]. The number of taxa in the phytoplankton community was significantly lower in spring than in summer and autumn [26]. Overall, phosphate was an important factor in the phosphate dependence of *Coscinodiscus radiatus*, *Melosira* sp., and *Odontella sinensis* in all seasons. The concentration of phosphorus, which has the greatest influence on plankton growth, was also a significant factor in relation to the composition of the plankton population [27,28]. Increased nutrient availability increases phytoplankton biomass and community composition [15,29], and seasonal phytoplankton biomass increases rapidly during austral spring [30]. Throughout the study period, diatoms predominated over other species in the phytoplankton community in the nearshore of the current study. To detect local and global changes, it is crucial to identify key species and monitor changes in species composition [31]. Centric diatoms were dominant over pennate diatoms. Izaguirre et al. reported that centric diatoms are one of the best-adapted algal groups and that phosphate enrichment may significantly benefit diatom growth in the initial spring phase and summer [32]. It is well known that stressed environments have fewer species, with only one or two species having significantly more individuals than the other species [33]. Oxygen is produced by the centric diatom *Coscinodiscus* sp., and there was a positive correlation between DO and the plankton community, which may be because these organisms consume CO_2_ and release O_2_, which raises DO.

### 3.2. Effect of Nutrients on Zooplankton Dynamics

Increasing species diversity is indirectly connected to the more effective utilization of light for supporting photosynthetic activity and nutrient sequestration; therefore, light limitation may be essential for zooplankton. Salinity is a limiting factor in living organisms’ distribution, changing into salinity by dilution and evaporation in coastal ecosystems, most likely acting on fauna [34]. In contrast, we observed a greater diversity of zooplankton species during spring than in other seasons, which was positively correlated with parameters such as salinity and inorganic nitrogen at site 8 (Figure 5c). The mean total zooplankton abundance peaked in the spring and then started to decline until the following winter [35]. Changes in nutrients will lead to species composition and community structure variations and even affect zooplankton, thus changing the diversity and stability of marine ecosystems [36]. *Paracalanus parvus*, *Microsetella norvegica*, *Penilia* sp., and *Tintinnopsis* sp. was controlled by spring silicate and phosphate (Figure 3c), while *Acrocalanus gibber* and *Oithona similis* were related to phosphate (Figure 3d and Figure 5d). In this study, the most abundant zooplankton species in all seasons were copepods (*Acrocalanus* sp. *Acrocalanus gibber*, *Oithona brevicornis*, *Paracalanus parvus*, *Acartia centrura*, *Microsetella norvegica*, *Oithona similis*). Several authors have reported that in many countries, the failure of fisheries is attributed to reduced zooplankton, especially copepod populations [37]. In this study, cyclopoid copepod *Oithona* species were observed in all seasons. Gallienne and Robins reported that *Oithona* is possibly an essential copepod in the world’s oceans [38]. The strong correlation between elevated nutrients (N, P) and specific diatom dominance highlights the impact of anthropogenic runoff from agricultural and municipal sources. This human-induced nutrient enrichment can trigger localized eutrophication, shift community compositions, and alter plankton diversity in these nearshore waters.

### 3.3. Copepod–Diatom Interactions

Copepod–diatom interactions provide insight into how grazers influence diatom population dynamics. Variations in polyunsaturated aldehyde (PUA) production can influence overall diatom abundance and reduce copepod egg-hatching success. These dynamics are further shaped by species-specific differences in copepod sensitivity, selective feeding behaviors, and the overall intensity and duration of field blooms [39]. In addition, diatoms are the most prevalent and dominant taxa in freshwater environments [40].

Amino acids also influence copepod food quality, sterols, proteins, vitamins, and fatty acids, which control copepod reproduction and growth [41,42]. Many studies have reported grazing selectivity against diatoms, while others have claimed that diatoms have nutritional superiority and that copepods strongly prefer diatoms [43,44]. In the present study, we found a link between grazing on diatoms by calanoid, cyclopoid, and harpacticoid copepods and the contribution of environmental parameters. The redundancy analysis (RDA) results showed that *Skeletonema costatum* and *Cyclotella* sp. were high in inorganic nitrogen, whereas *Paracalanus parvus* and *Oithona similis* were also observed during autumn. There was a relationship between *Paracalanus parvus* and *Thalassionema nitzschioides* in high silicate during winter. *Acartia centrura* commonly occurs in estuaries, grows fast, and breeds continuously, with high reproductive capacity. During summer, salinity exerted a strong influence on the distribution of the diatoms *Thalassiothrix longissima* and *Coscinodiscus* sp., which occurred alongside changes in the abundance of the copepod *Acartia centura*. In contrast, phosphate was influenced by both *Microsetella norvegica* and *Melosira* sp. during the spring season. These findings have proven that copepods do not graze on all kinds of diatoms. Diatoms are often a significant food source for zooplankton, and mesozooplankton are critical phytoplankton consumers [45]. They play an essential role in the oceanic biogeochemical cycle of carbon and other elements [46]. It has been associated with productive pelagic food chains, leading to planktonic copepod suspension feeding in top consumers and fisheries [47].

Diatoms are thought to be the most important food source for copepods in the sea, and they have a high nutritional value for organisms that graze on them [48]. Although diatoms are generally a good food source for grazers, recent research has found that some species have chemical defenses. The secondary metabolites that these algae immediately release after being injured are not intended to harm the predators directly but rather to hinder their ability to reproduce [49]. Diatoms can alter the marine food web using this tactic to lower the number of grazers [50]. Several studies have revealed that feeding copepods monospecific diatom diets reduces hatching success [51,52]. We observed the interaction of diatoms and copepods in different seasons and environmental parameters in the current study. During autumn, diatoms such as *Skeletonema costatum*, *Cheatoceros decipiens*, and *Cyclotella* predominated, while the copepod *Paracalanus parvus* was also abundant. During the winter, *Ditylum brightwellii* and *Odontella sinensis* were abundant, whereas *Paracalanus parvus* was dominant. The phytoplankton communities *Cheatoceros*, *Melosira*, *Thalassionema nitzschioides*, *Coscinodiscus centralis*, and *Ditylim sol* were dominant in this study, while *Microsetella norvegica*, *Penilia* sp. *Paracalanus parvus*, and *Tintinnopsis* were abundant during spring. During the summer, *Thalassiothrix longissima*, *Coscinodiscus*, and *Ditylum brightwellii* were dominant, whereas *Acrocalanus gibber*, *Acartia centrura*, *Tintinnopsis*, and *Oithona brevicornis* were dominant. Our research leads us to conclude that the dominant copepod species that graze on diatom abundance described in this study will be helpful for the cultivation of copepods with selective feeds in the future. Diatoms are significant phytoplankton that contribute significantly to the primary productivity of marine ecosystems [53], with up to 40% of the ocean’s annual primary production coming from them [54] and 25% of the world’s carbon being fixed by them [55].

### 3.4. Cultivation of Microalgae and Copepods as Potential Applications

The predominance of diatoms and copepods as well as the environmental factors observed in this study will be used to cultivate microalgae for a variety of potential applications. Secondary metabolites in microalgae include astaxanthin, omega-3 fatty acids, sterols, proteins, enzymes, vitamins, and pigments [56]. Furthermore, microalgae contain carbohydrates, proteins, and other nutrients necessary for animal growth, and they have been studied for use as animal feed [57]. Researchers have become interested in microalgae extracts because they have demonstrated anticancer (antiproliferative) activities in a variety of cancer types [58]. Fuel consumption and pollution are at their highest levels ever today, and if they continue at this rate, we will face significant ecological problems in the near future [59]. Microalgae would aid in increasing lipid accumulation, allowing it to be used in diatom solar panels to produce DiafuelTM [60]. There are a number of significant and novel parameters that should be carefully examined to advance copepod cultivation methods. Copepods account for up to 71% of the mesozooplankton biomass [61], and they are a vital source of food for planktivorous fish and fish larvae in general [62]. Docosahexaenoic acid (DHA) and eicosapentaenoic acid (EPA), which account for 60% of all fatty acids, are present in high concentrations in a number of coastal copepods [63]. Astaxanthin, a natural antioxidant, and vitamins C and E are all found in copepods [64]. Overall, these seasonal successions show that the community responds to broader hydrological shifts rather than to isolated environmental factors alone.

## 4. Materials and Methods

### 4.1. Study Area

**Figure 7:**
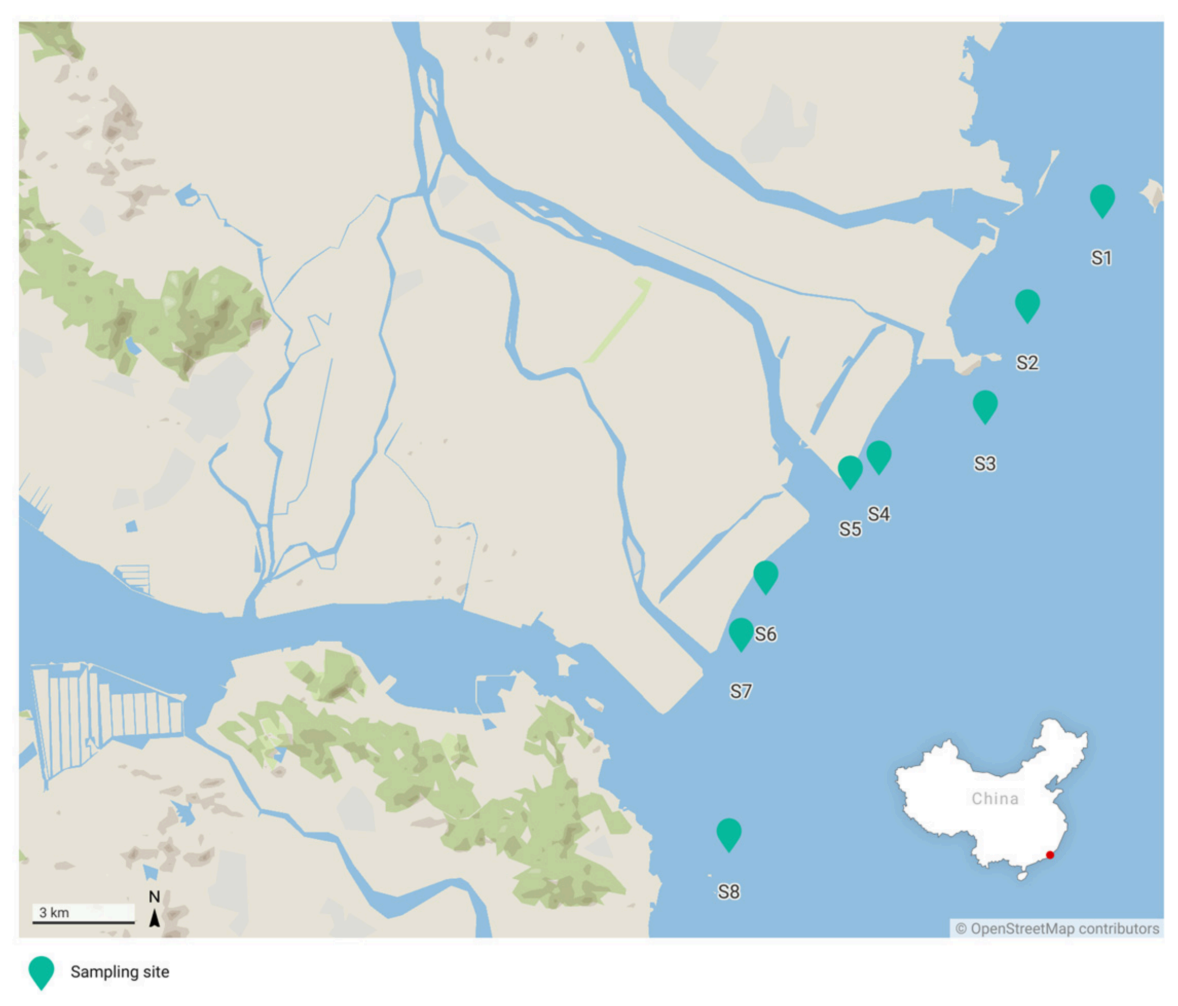
Study area and the locations of the sampling sites in the nearshore waters in Eastern Guangdong, Southeast China.

The field study was carried out in the nearshore area of the Hanjiang River (23.45 N, 116.88 E) in Eastern Guangdong, China (Figure 7). The Hanjiang River is the primary drinking water source for the cities of Chaozhou and Shantou in Guangdong Province, Southern China. The main body of the Hanjiang River can be up to 470 km long. As it enters the South China Sea in the Chenghai and Longhu Districts of Shantou, the Hanjiang River flows through the Han River Delta to the south. The average annual rainfall and runoff for the Hanjiang River are 2033 mm and 3.5 billion m^3^, respectively (Guangdong Provincial Water Resources Department, 2015). The population density along the Hanjiang River leads to significant outflows of residential sewage [1].

### 4.2. Determination of Environmental Variables

Plankton samples were collected from eight sampling sites during autumn (November), winter (February), spring (May), and summer (August) using a phytoplankton net (0.35 m; mesh size 48 µm) and a zooplankton net (115 µm). Separately, discrete surface water samples were collected at each site using a 1 L sampling bottle for environmental and nutrient analyses. Surface seawater temperature (T) was measured using a centigrade thermometer, salinity (S) was estimated using a hand refractometer (ATAGO PAL-03S, Atago, Tokyo, Japan), and pH was recorded with a pH meter (Model-LC-120, Elico, Telangana, India). Dissolved oxygen (DO) was determined via the Winkler titration method. For dissolved nutrients, the collected water samples were filtered using a 0.45 µm Millipore system, stored at −20 °C, and analyzed spectrophotometrically for inorganic nitrogen (N), phosphate (P), and reactive silicate (Si) following standard APHA colorimetric protocols.

### 4.3. Plankton Identification and Enumeration

Plankton samples were collected at each station by towing a plankton net equipped with a flow meter. The concentrated collections (approximately 1 L each) were preserved in 5% neutralized formalin for quantitative analysis. A 0.1 mL subsample was examined in a Sedgwick–Rafter counting cell using an inverted light microscope (Axio Observer 3, Carl Zeiss, Oberkochen, Germany), while zooplankton identification followed conventional keys [65]. Phytoplankton density was expressed as cells·m^−3^, while zooplankton density was calculated as individuals·m^−3^ [66]. Taxonomic identification and volumetric quantification for phytoplankton followed standard protocols [67].

### 4.4. Data Analyses

Biodiversity indices such as species diversity, richness, evenness, and dominance were calculated using standard formulae [68] and the relative dominance (Y) was calculated as the product of the ratio of the number of individual species to the total number of individuals of all species present in the sample and the frequency of the species at the sampling location. Dominant species had a Y value greater than 0.02. Seasonal variations in environmental variables were assessed using ChiPlot (https://chiplot.online). The study applied Spearman’s rank correlation using XLSTAT V.2016 software to analyze the relationship between the environmental variables and the dominance of phytoplankton and zooplankton. Gradient length results obtained using detrended correspondence analysis (DCA) (OriginPro 2023, OriginLab, Northampton, MA, USA) were less than 3.0 for the response data, suggesting there were short environmental gradients in the study site over the four seasons (Table 1). Hence, RDA for diversity and ecological parameters was performed using the OmicStudio tools (https://www.omicstudio.cn/tool (accessed on 30 July 2026); based on R version 3.6.3) following the standardization of the plankton densities. The vegan package 2.4-3 in R-3.4.0 software was used for plankton abundance calculations. Table 2 and Table 3 show the adjusted R^2^, F, and significances.

### 4.5. Limitations and Future Perspectives

While this study provides an important ecological baseline for the Hanjiang River’s nearshore area, it has numerous limitations. First, our sampling design was restricted to distinct seasonal snapshots, which may overlook high-frequency plankton successions caused by intense episodic monsoon rainfall or abrupt shifts in the delta’s 3.5 billion m^3^ annual runoff. Second, our morphological counts are predominantly based on dominating functional groups, and unusual or cryptic picoplankton lineages require further molecular validation. Future research should prioritize continuous, high-frequency spatial tracking across the Hanjiang River Delta. This tracking should explicitly link micro-ecological monitoring to long-term quantification of residential sewage loads and localized land-use shifts. Such data will allow researchers to estimate how rising coastal urbanization affects subtropical plankton stability.

## 5. Conclusions

Our findings demonstrate that localized environmental variables heavily regulate the seasonal distribution, diversity, and dominance profiles of nearshore plankton communities. Throughout the study period, 62 phytoplankton species and 32 zooplankton species were documented, revealing a seasonal contraction in taxonomic variety and species evenness across specific sampling stations. Redundancy analysis identified distinct, community-specific hydrographic drivers. Phytoplankton abundance and succession patterns were primarily driven by shifting nutrient loads (inorganic nitrogen and phosphate), salinity, and pH gradients. Conversely, zooplankton community responses were heavily shaped by variations in water temperature, salinity, and phosphate levels. These field results establish a critical ecological baseline for nearshore coastal health in Eastern Guangdong, accurately tracking how seasonal hydrographic transitions and localized nutrient enrichment directly dictate the structural baseline of subtropical marine food webs.

## Figures and Tables

**Table 1:** List of the relative dominance of the identified phytoplankton species across the four seasons.

No	Species Name	Short Name	Autumn	Winter	Spring	Summer
1.	*Amphora* sp.	*Amp*	0.000	0.000	0.007	0.000
2.	*Asterionellopsis glacialis*	*Ast_g*	0.012	0.000	0.015	0.000
3.	*Bacillaria paxillifera*	*Bac_p*	0.000	0.009	0.017	0.006
4.	*Bacteriastrum hyalinum*	*Bac_h*	0.003	0.010	0.009	0.010
5.	*Bacteriastrum varians*	*Bac_v*	0.001	0.000	0.012	0.000
6.	*Ceratium tripos*	*Cer_t*	0.000	0.000	0.008	0.000
7.	*Ceratium furca*	*Cer_f*	0.037	0.014	0.019	0.006
8.	*Ceratium tricoceros*	*Cer_tr*	0.000	0.000	0.011	0.000
9.	*Ceratium horridum*	*Cer_h*	0.001	0.000	0.003	0.002
10.	*Chaetoceros* sp.	*Cha*	0.039	0.031	0.039	0.002
11.	*Chaetoceros decipiens*	*Cha_d*	0.020	0.015	0.020	0.000
12.	*Chaetoceros curvisetus*	*Cha_c*	0.000	0.000	0.000	0.006
13.	*Chaetoceros lorenzianus*	*Cha_l*	0.005	0.020	0.029	0.000
14.	*Chaetoceros tortissimus*	*Cha_t*	0.000	0.002	0.000	0.000
15.	*Chlorella* sp.	*Chl*	0.000	0.000	0.023	0.000
16.	*Coscinodiscus gigas*	*Cos_g*	0.001	0.000	0.002	0.000
17.	*Coscinodiscus oculus-iridis*	*Cos_o*	0.000	0.001	0.014	0.004
18.	*Coscinodiscus granii*	*Co_gr*	0.027	0.000	0.002	0.000
19.	*Coscinodiscus centralis*	*Cos_c*	0.001	0.023	0.022	0.001
20.	*Coscinodiscus* sp.	*Cos*	0.130	0.058	0.000	0.105
21.	C. *asteromphalus*	*Cos_a*	0.003	0.000	0.000	0.001
22.	*Coscinodiscus radiatus*	*Cos_r*	0.026	0.004	0.001	0.000
23.	*Corethron* sp.	*Cor*	0.000	0.002	0.000	0.007
24.	*Cyclotella* sp.	*Cyc*	0.152	0.013	0.000	0.001
25.	*Closterium* sp.	*Clo*	0.000	0.000	0.005	0.000
26.	*Ditylim sol*	*Dit_s*	0.046	0.078	0.028	0.060
27.	*Dinophysis caudata*	*Din_c*	0.001	0.000	0.000	0.000
28.	*Ditylum brightwellii*	*Dit_b*	0.070	0.097	0.083	0.106
29.	*Eucampia zodiacus*	*Euc_z*	0.000	0.000	0.003	0.000
30.	*Fragilaria* sp.	*Fra_s*	0.000	0.000	0.000	0.001
31.	*Guinardia flaccida*	*Gun_s*	0.000	0.000	0.000	0.002
32.	*Hemiaulus* sp.	*Hel*	0.000	0.000	0.000	0.002
33.	*Hemiaulus sinensis*	*Hem_si*	0.000	0.000	0.000	0.002
34.	*Helicotheca* sp.	*Hel_s*	0.000	0.038	0.000	0.000
35.	*Licmophora* sp.	*Lic_s*	0.000	0.001	0.000	0.000
36.	*Leptocylindrus danicus*	*Lep_d*	0.004	0.000	0.009	0.000
37.	*Melosira granulate*	*Mel_g*	0.000	0.000	0.001	0.000
38.	*Melosira sulcata*	*Mel_s*	0.000	0.000	0.004	0.000
39.	*Melosira* sp.	*Mel*	0.000	0.013	0.023	0.015
40.	*Navicula* sp.	*Nav*	0.000	0.001	0.003	0.000
41.	*Nitzchia* sp.	*Nit*	0.000	0.004	0.010	0.000
42.	*Odontella sinensis*	*Odo_s*	0.000	0.033	0.006	0.010
43.	*Oscillatoria* sp.	*Osc*	0.000	0.009	0.001	0.000
44.	*Odontella mobiliensis*	*Odo_m*	0.000	0.062	0.005	0.026
45.	*Pinnularia* sp.	*Pin*	0.000	0.000	0.003	0.000
46.	*Pleurosigma elongatum*	*Ple_e*	0.000	0.000	0.002	0.002
47.	*Pleurosigma* sp.	*Plu_s*	0.000	0.001	0.000	0.000
48.	*Periastrum simplex*	*Per_s*	0.000	0.001	0.008	0.000
49.	*Pediastrum* sp.	*Ped*	0.000	0.000	0.001	0.000
50.	*Rhizosolenia setigera*	*Rhi_s*	0.000	0.002	0.001	0.000
51.	*Rhizosolenia styliformis*	*Rhi_st*	0.000	0.000	0.002	0.015
52.	*Rhzhisolenia simplex*	*Rh_si*	0.000	0.000	0.005	0.000
53.	*Rhizosolenia* sp.	*Rhiz_s*	0.029	0.003	0.000	0.002
54.	*Surirella* sp.	*Sur*	0.000	0.000	0.003	0.000
55.	*Skeletonema costatum*	*Ske_c*	0.036	0.010	0.044	0.195
56.	*Stephanopyxis turris*	*Ste_t*	0.000	0.001	0.003	0.000
57.	*Synedra* sp.	*Syn*	0.000	0.000	0.014	0.000
58.	*Triceratium* sp.	*Tri_s*	0.000	0.001	0.000	0.000
59.	*Thalassiosira* sp.	*Tha*	0.010	0.006	0.040	0.013
60.	*Thalassiosira lineata*	*Th_l*	0.000	0.020	0.002	0.000
61.	*Thalassionema nitzschioides*	*Tha_n*	0.001	0.024	0.020	0.109
62.	*Thalassiothix longissima*	*Tha_l*	0.035	0.001	0.006	0.024

**Table 2:** A list of the relative dominance of the identified zooplankton species over the four seasons.

No	Species Name	Short Name	Autumn	Winter	Spring	Summer
1.	*Acartia centrura*	*Aca_c*	0.002	0.008	0.007	0.028
2.	*Acartia* sp.	*Aca_s*	0.002	0.002	0.000	0.005
3.	*Acantho cyclops*	*Aca_cy*	0.000	0.000	0.001	0.000
4.	*Acrocalanus gibber*	*Acr_g*	0.002	0.002	0.001	0.020
5.	*Acrocalanus* sp.	*Acr_s*	0.002	0.091	0.000	0.001
6.	*Bestiolina amoyensis*	*Bes_a*	0.002	0.000	0.001	0.000
7.	*Centropages* sp.	*Cen*	0.002	0.007	0.002	0.000
8.	*Centropyxis*	*Cen_y*	0.000	0.006	0.006	0.000
9.	*Copepod larva*	*Cop_n*	0.139	0.142	0.127	0.143
10.	*Codonella* sp.	*Cod*	0.000	0.000	0.018	0.000
11.	*Corycaeus affinis*	*Cor_a*	0.017	0.000	0.001	0.005
12.	*Codonellopsis morchella*	*Cod_m*	0.000	0.000	0.000	0.002
13.	*Cirripedia larva*	*Cir_n*	0.000	0.000	0.000	0.025
14.	*Cyclops* sp.	*Cyc_s*	0.000	0.000	0.000	0.002
15.	*Ecocyzicus* sp.	*Eo_sp*	0.000	0.000	0.011	0.001
16.	*Euterpina acutifrons*	*Eut_a*	0.008	0.000	0.000	0.005
17.	*Favella* sp.	*Fav_s*	0.267	0.000	0.000	0.012
18.	*Gastropod larva*	*Gas_l*	0.000	0.000	0.002	0.002
19.	*Leprotintinnus* sp.	*Lep_s*	0.008	0.017	0.000	0.059
20.	*Labidocera euchaeta*	*Lab_e*	0.000	0.001	0.000	0.000
21.	*Lucifer* sp.	*Luc_s*	0.002	0.000	0.000	0.000
22.	*Microsetella norvegica*	*Mic_n*	0.000	0.001	0.028	0.008
23.	*Nannocalanus* sp.	*Nan_s*	0.003	0.006	0.000	0.004
24.	*Oithona brevicornis*	*Oit_b*	0.002	0.089	0.000	0.024
25.	*Oithona similis*	*Oit_s*	0.040	0.079	0.000	0.003
26.	*Oithona attenuata*	*Oit_a*	0.000	0.008	0.000	0.000
27.	*Paracalanus* sp.	*Par_s*	0.004	0.012	0.000	0.002
28.	*Paracalanus parvus*	*Par_p*	0.146	0.107	0.111	0.042
29.	*Paracyclops* sp.	*Pac_s*	0.000	0.000	0.000	0.001
30.	*Penilia* sp.	*Pen_s*	0.000	0.028	0.038	0.000
31.	*Polychaeta larva*	*Pol_l*	0.000	0.001	0.028	0.000
32.	*Tintinnopsis* sp.	*Tin_s*	0.028	0.008	0.077	0.054
33.	*Tintinnopsis radix*	*Tin_r*	0.000	0.000	0.002	0.170
34.	*Tintinnopsis sulfata*	*Tit_s*	0.000	0.000	0.000	0.001

**Table 3:** Redundancy analysis (RDA) and seasonal Spearman’s rank correlation statistics between environmental factors and plankton communities.

Season	Axis	Eigenvalue		Correlation Between Phytoplankton and Zooplankton
		Phytoplankton	Zooplankton	0.714
Autumn	Axis1	0.107	0.246	
	Axis2	0.040	0.181	
	Axis3	0.041	0.152	
	Axis4	0.411	0.173	
Winter	Axis1	0.146	0.392	0.810
	Axis2	0.474	0.244	
	Axis3	0.046	0.178	
	Axis4	4.688	0.230	
Spring	Axis1	0.262	0.231	0.857
	Axis2	0.137	0.055	
	Axis3	0.098	0.102	
	Axis4	0.030	0.103	
Summer	Axis1	0.174	0.328	0.429
	Axis2	0.095	0.131	
	Axis3	0.068	0.151	
	Axis4	0.099	0.841	

## Data Availability

The datasets generated for this study can be found in the Figshare repository (https://doi.org/10.6084/m9.figshare.21997712).
